# A dimensional perspective on the genetics of obsessive-compulsive disorder

**DOI:** 10.1038/s41398-021-01519-z

**Published:** 2021-07-21

**Authors:** Nora I. Strom, Takahiro Soda, Carol A. Mathews, Lea K. Davis

**Affiliations:** 1 Institute of Psychiatric Phenomics and Genomics (IPPG), University Hospital, LMU Munich, Munich, Germany; 2 Department of Clinical Neuroscience, Karolinska Institutet, Stockholm, Sweden; 3 Department of Biomedicine, Aarhus University, Aarhus, Denmark; 4 Department of Psychology, Humboldt Universität zu Berlin, Berlin, Germany; 5 Department of Psychiatry, Duke University, Durham, NC USA; 6 Department of Psychiatry, University of Florida, Gainesville, FL USA; 7 Division of Genetic Medicine, Department of Medicine, Vanderbilt University Medical Center, Nashville, TN USA; 8 Department of Psychiatry and Behavioral Sciences, Vanderbilt University Medical Center, Nashville, TN USA; 9 Vanderbilt Genetics Institute, Vanderbilt University Medical Center, Nashville, TN USA

**Keywords:** Genetics, Psychology

## Abstract

This review covers recent findings in the genomics of obsessive-compulsive disorder (OCD), obsessive-compulsive symptoms, and related traits from a dimensional perspective. We focus on discoveries stemming from technical and methodological advances of the past five years and present a synthesis of human genomics research on OCD. On balance, reviewed studies demonstrate that OCD is a dimensional trait with a highly polygenic architecture and genetic correlations to multiple, often comorbid psychiatric phenotypes. We discuss the phenotypic and genetic findings of these studies in the context of the dimensional framework, relying on a continuous phenotype definition, and contrast these observations with discoveries based on a categorical diagnostic framework, relying on a dichotomous case/control definition. Finally, we highlight gaps in knowledge and new directions for OCD genetics research.

## Introduction

Obsessive-compulsive disorder (OCD) is a chronic, neuropsychiatric condition with an estimated lifetime prevalence between 1% and 3% [1, 2]. It is characterized by obsessions and compulsions that are time consuming and cause clinically significant distress or impairment in important areas of functioning. Obsessions are intrusive repetitive thoughts, urges, or mental images that are difficult to control, often do not serve a purpose and are accompanied by negative mood or distress. Compulsions are repetitive behaviors that an affected person feels compelled to perform repeatedly, thereby attempting to reduce the distress caused by the obsessions or to prevent dreaded events. Avoidance of situations that can trigger obsessions can also occur [3–6]. The age of onset of OCD symptoms is bimodal. While most individuals develop OCD symptoms in childhood (on average, around age 10), some have a later age of onset, with symptoms developing during adolescence or young adulthood (on average, around age 21) [7–9]. While this review focuses on OCD among adults, it is important to consider that symptom subtypes, distress, and insight into symptoms differ across development, and in early development (between ages 2 and 5) obsessive-compulsive symptoms are normal and ubiquitous [10, 11]. Finally, while the sex ratio is approximately 1:1 among adults with OCD, males are somewhat more likely to exhibit the childhood-onset form than females (male:female ratio between 2:1 and 3:1) [12, 13].

Genetic investigations of OCD typically apply a conceptual framework that relies on either clinical diagnosis [14] (i.e., Diagnostic Conceptual Framework), based on a dichotomous case/control definition; or a dimensional framework [6, 15, 16] (i.e., Dimensional Conceptual Framework), based on a continuous phenotype definition. The study of OCD genetics has largely relied on the more traditional Diagnostic Conceptual Framework while more recently the study of obsessive-compulsive symptoms (OCS) in the general population has employed the Dimensional Conceptual Framework. Importantly, these two frameworks are not mutually exclusive, although they differ in terms of their application to genomic study designs, underlying assumptions, advantages, and limitations (Table 1).

**Table 1:** Diagnostic and dimensional frameworks.

Conceptual framework	GWAS approach	Assumptions	Advantages	Limitations
**Diagnostic** (e.g., leverages the discriminatory qualities of a clinical diagnosis to distinguish biologically meaningful differences between two groups of people)	GWAS of clinically ascertained cases vs controls	Oversampling of phenotypic extremes will improve power to detect associated genetic variants	Reduces heterogeneity Knowledge gained is directly applicable to a clinical population	May overestimate effect sizes Impairment or distress is inherently ascertained which may limit generalizability
**Dimensional** (e.g., leverages the continuous nature of psychiatric symptoms in clinical or non-clinical populations to identify biologically meaningful contributions to behavior)	GWAS of symptom characteristics in the whole population (e.g., symptom counts, level of impairment or distress, etc.)	Subclinical symptoms share the same genetic correlates as diagnosed OCD	Increased opportunity for participation and increased sample size Knowledge gained is generalizable to a broader population	Increases phenotypic and genetic heterogeneity Findings may have less immediate clinical relevance

**Fig. 1:**
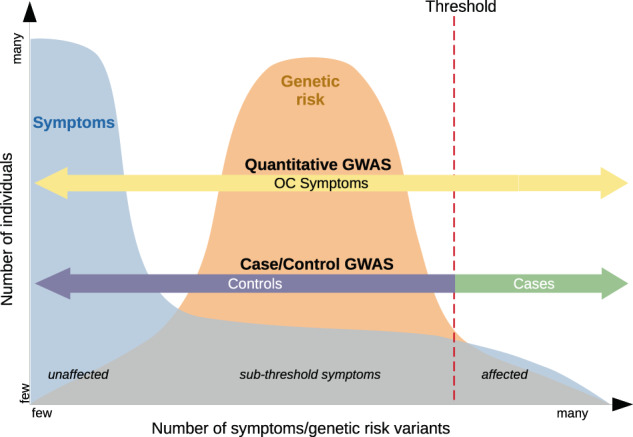
Liability threshold model. In orange the distribution of genetic-risk variants for OCD in the population from few to many. In blue the distribution of OC symptoms in the population. The red dashed line depicts the diagnostic threshold. In a case/control GWAS (diagnostic framework) all individuals to the left (purple arrow) of the threshold would be considered a control, while all individuals to the right (green arrow), with a clinical diagnosis of OCD, would be considered a case. In a quantitative GWAS (dimensional framework) all individuals across the symptom- and genetic-risk-spectrum (yellow arrow) would be included, also weighting in subthreshold symptoms.

**Fig. 2:**
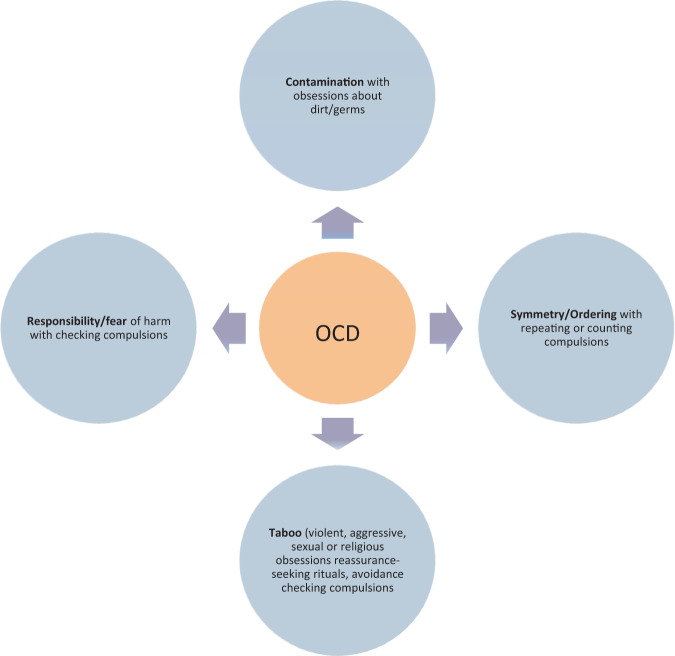
OCD symptom sub-types. OCD symptoms are classically defined by four subtypes, including (1) responsibility/fear of harm (obsessions regarding responsibility and checking compulsions such as asking for reassurance or checking that harm did not occur to someone), (2) contamination (obsessions about dirt or germs or other possible contaminants and cleaning compulsions), (3) symmetry/ordering (obsessions about symmetry or order and ordering, repeating or counting compulsions), and (4) taboo (violent, aggressive, sexual or religious obsessions with reassurance-reeking rituals, avoidance or checking for harm) [1–4].

**Fig. 3:**
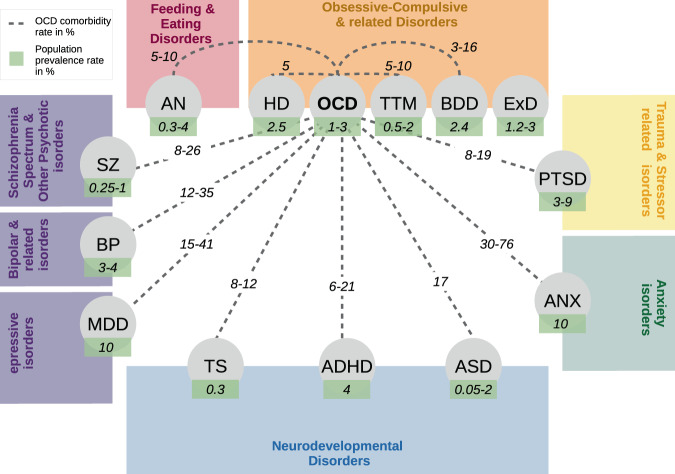
Phenotypic associations between OCD and other psychiatric disorders, grouped by their DSM-5 categorization. Comorbidity estimates between OCD and AN [95, 96], TS [88, 89], HD [128], TTM [91, 100], BDD [91, 100, 129], ExC [62, 109], PTSD [1, 100], ANX [1, 91], MDD [1, 91, 92], ASD [97], ADHD [1, 91, 99, 100], BP [100, 130], and SZ [130] are on the dashed lines, while the green boxes indicate the population prevalence of each disorder (OCD [2]; AN [131]; TS [132]; HD [82]; TTM [85, 86]; BDD [83, 84]; ExD [87, 105, 133]; PTSD [90, 134, 135]; ANX [1, 90]; MDD [1, 90]; ASD [97, 98]; ADHD [90]; BP [136, 137]; and SZ [138, 139]).

Approaches to identify genes associated with OCD typically take advantage of these frameworks and often attempt to either narrow the phenotypic definition to reduce heterogeneity or broaden the phenotypic definition to leverage observed genetic or clinical relationships between OCD and other traits. Methodologies aimed at narrowing the phenotypic definition may use a strict case definition or only include cases with clinically diagnosed OCD [1, 17, 18], childhood-onset OCD [4, 7], OCD from multiplex families [19, 20], OCD with tics [21], severe OCD, or specific OC symptoms [22] (Fig. 1). On the other hand, studies that broaden the phenotype may include subclinical OCS [23–25] (Fig. 2) or closely related psychiatric disorders [12] (Fig. 3). The advantages of this type of model primarily relate to potentially increased power due to larger available sample sizes. Although dimensional approaches are traditionally population-based, it should be noted that dimensional approaches can be used in clinically ascertained samples as well.

Here we review genetic evidence obtained using approaches that employ both a diagnostic (case/control) and a dimensional (continuous phenotype) framework and discuss the implications for future genetic research of OCD. We only briefly address the genetic findings from studies using the diagnostic conceptual framework in OCD, as recent reviews have discussed these in depth [26–29]. The majority of this review focuses instead on recent advances in OCD genomics research by examining the evidence for genetic correlates of OCD using a dimensional perspective. The primary emphasis is on findings from genome-wide research but will also draw on data arising from other methodologies (twin- and family studies) where genome-wide data is not yet available.

### Key concepts and definitions

#### Dimensionality

Refers to the concept of phenotypic variation which lies along a continuum from no symptoms through subclinical symptoms to severe and impairing symptoms.

#### Complex trait liability threshold model

This model assumes that there is an underlying distribution of genetic risk in the general population for OCD and other complex traits that contributes to varying levels of symptom expression, with clinically recognized disease at one end of the distribution [30].

#### OCD symptom subtypes

OCD is phenotypically heterogeneous, and different individuals can express distinct sets of characteristic symptoms. The different symptom subtypes may have diverse underlying biological disease mechanisms, depending on symptom characteristics, severity, etc. Creating subgroups of symptoms that commonly co-occur for genetic and other studies may decrease the underlying heterogeneity and facilitate gene-finding efforts.

#### OCRD

OCD shares certain characteristics, such as intrusive thoughts and/or repetitive behaviors with other related disorders such as hoarding disorder, body dysmorphic disorders, excoriation disorder, and trichotillomania. Based on these phenotypic similarities, these disorders are categorized as obsessive-compulsive and related disorders (OCRDs) in the DSM-5 [14].

#### Genome-wide association study (GWAS)

An approach used in genetics research to associate specific genetic variations with disease but cannot on its own specify which genes are causal. GWAS approaches usually search for single nucleotide polymorphisms (SNPs) across the entire genome that are consistently associated with the trait of interest. Traditionally, the allele frequencies of affected vs. unaffected individuals are compared but quantitative phenotypic data can also be analyzed [31].

#### Cross-disorder GWAS

A cross-disorder GWAS aims to identify cross-phenotype genetic influences that transcend diagnostic boundaries by including genetic data from various disorders into one GWAS. This approach can identify SNPs with pleiotropic effects, meaning that they influence more than one distinct phenotypic trait or a common mechanism underlying more than one disorder.

#### Heritability

Heritability refers to the proportion of trait variation in a population that can be explained by variation genotypes in that population.

#### Genetic correlation

Genetic correlation is the proportion of variance that two traits share due to genetic causes. It arises from the pleiotropic effects of genes on multiple traits or through linkage disequilibrium between distinct loci that each affect one trait. Traits can be either positively correlated (meaning that the underlying genetic variants contribute to increased risk of both traits), or negatively correlated (meaning that the underlying genetic variants contribute to increased risk of one trait and decreased risk of the other).

#### Polygenic risk score

A polygenic (risk) score is a value that summarizes the estimated effect of many genetic variants on an individual’s phenotype, typically calculated as a weighted sum of trait-associated alleles [32, 33].

#### GenomicSEM

Genomic Structural Equation Modeling is a multivariate method that can be used to model the joint genetic architecture of complex traits, identify SNPs with effects on a common factor of cross-trait liability and identify SNPs that cause divergence between traits [34].

### The genetic architecture of OCD

OCD has a multifactorial etiology and results from a combination of genetic and environmental impacts across the lifespan [7, 35–37]. The earliest evidence for a genetic component to the etiology of OCD arose from family and twin studies. Rates of both OCD and subclinical obsessional symptoms are higher (4- to 20-fold increased risk) in family members of individuals with OCD than in family members of unaffected controls [19, 38–41]. Twin studies in both clinical and population-based samples also demonstrate a much higher concordance of OCD symptoms among monozygotic twins than dizygotic twins providing the earliest assessments of the heritability of OCD ranging from 0.29 to 0.58 [9, 16, 20, 42–51], while the childhood-onset form of OCD is consistently found to have a higher heritability than the adult-onset form [52].

Modern genomic methods demonstrated that the genetic contribution to OCD is primarily polygenic, meaning that hundreds or thousands of genetic variants each contribute a very small amount to the overall genetic predisposition to OCD [50, 53]. There is some evidence from copy number variants studies and whole-exome sequencing studies that rare variants exerting larger effects may also play a role in OCD development in a subset of individuals or families [54–59], but less work has been done in this area. The genetic liability (i.e., aggregate effect of risk alleles) to OCD can be modeled as a normal continuous distribution across the population, with few people carrying very few risk alleles, few people carrying very many risk alleles, and most people somewhere in the middle of the distribution. The threshold (t) along this complex trait liability distribution, beyond which an individual is diagnosed, is a function of the population prevalence (K) of OCD diagnosis (Fig. 1; genetic-risk curve). Genome-wide association studies (GWAS) [31] of clinically ascertained OCD suggest that the degree of heritability directly attributable to measured genetic variants (e.g., SNP or genomic heritability) is around 23% (SE = 0.04) [17, 18, 60]. To date, GWAS studies of OCD remain underpowered and no individual SNPs or genes with replicable genome-wide significant findings have been identified, although work is underway to increase sample sizes and potential for SNP discovery.

#### OCD symptom subtypes

While OCD patients vary widely with respect to a range of factors (e.g. symptom severity, age of symptom onset, comorbidities), symptom type has been most extensively discussed as a source of potential phenotypic and genetic heterogeneity. It is hypothesized that each symptom subtype dimension may be associated with a distinct set of underlying psychological and biological mechanisms, however, it remains unclear whether these clinical subtypes represent genetic subtypes as well [5, 6, 61]. It is also possible that OCD symptoms, independent of subtype or clinical presentation, are influenced by a general underlying genetic susceptibility to OCD either alone or in combination with genetic or environmental contributors to specific symptom groups.

Several studies have used data reduction techniques such as factor analyses to identify symptom-specific OCD subtypes [5, 6, 15] as discussed in Cullen et al., Pinto et al. and reviewed in Bloch et al., Pauls et al. [4, 35, 62, 63]. OCD symptoms are classically defined by four subtypes including (1) responsibility/fear of harm, (2) contamination, (3) symmetry/ordering, and (4) taboo (see Fig. 2) [3–6]. Hoarding obsessions and compulsions and somatic obsessions and body-related checking compulsions may also be OCD symptoms, although these may also be indicative of distinct disorders (hoarding disorder and body dysmorphic disorder or an illness anxiety disorder, respectively). One limitation of these analyses is that the majority parse OCD symptomatology by clustering symptoms that tend to occur together, as in factor or cluster analyses, rather than by clustering individuals with similar presentations together, as in latent class analysis. To date, only one latent class analysis of OCD has been conducted [64]. This study found that, rather than being classified by specific symptom types, individuals with OCD were more often clustered according to symptom severity, perhaps providing additional support for a severity spectrum, but with less clear utility for symptom sub-phenotyping.

Family and twin studies have reported mixed results regarding the heritability of specific OC symptom dimensions [5, 16, 43, 51, 63, 65–69]. Some studies found all symptom factors to be heritable, but to varying degrees [5, 63, 69], while others reported high and significant heritability estimates only for some symptom factors, but not for others [24, 70]. The strength of reported genetic correlations between each of the individual symptom-dimensions varies, as does the genetic correlation between the symptom factors and OCD, possibly indicating the presence of both common and distinct biological underpinnings to these OCD symptom dimensions. Other studies have suggested that an underlying unidimensional construct not specific to symptom subgroups is a better fit for the available data [65, 70]. Mathews et al. [65] reported a core group of symptoms underlying obsessionality that was highly heritable and correlated with OCD, whereas the symptom factors that they extracted did not show significant heritability estimates. Their findings suggest that genetic factors may primarily contribute to the presence, and perhaps the severity, of obsessions, rather than to the specific type of symptom present. Similarly, van Grootheest et al. [70] found a common OC behavior phenotype that explained the variance in all three dimensions that they extracted (contamination, rumination, checking), with additional independent genetic influences acting on the contamination dimension.

Longitudinal symptom stability provides another lens through which to examine the genetic and environmental contributions to OCD and OC symptoms. Multivariate analyses of the Padua Inventory Revised assessed in 2002 and again in 2008 in the population-based Netherlands Twin Register indicate that OC symptoms are longitudinally stable in adults [71]. Longitudinal OC symptom stability was explained equally by both genetic (additive *r* = 0.56, and non-additive *r* = 1) and environmental factors (*r* = 0.43) [71]. Among children, clinical symptom presence may be influenced by developmental stages and some degree of ritualistic, hoarding, or arranging behavior is developmentally appropriate depending on the child’s age. Few studies have examined symptom stability in children. One such study including 74 children who were diagnosed with OCD, examined symptom subtypes over time using the Children’s Yale-Brown Obsessive-Compulsive Scale (CY-BOCS). Multiple regression analyses demonstrated that symptom domain at baseline was most predictive of symptom domain at follow-up, however, the partial correlations were modest and the ‘symmetry’ domain at baseline was predictive of both symmetry and hoarding at follow-up [72]. These findings also emphasize the contributions of genetic, environmental, and developmental influences on symptom subtype.

### Genetics of obsessive-compulsive symptoms in the general population

A corollary of the liability threshold model previously described, which assumes an underlying normal distribution of genetic risk in the general population, is that it contributes to varying levels of symptom expression, from no symptoms to subclinical symptoms to severe and impairing symptoms (see Fig. 1). Indeed, obsessive-compulsive symptoms (OCS) are relatively common in the general adult population and even more common during development [73]. However, while 13 to 38% of adults experience OCS, only 2–3% of these individuals meet criteria for a formal OCD diagnosis [6, 10, 15, 74]. Although the majority of individuals who endorse OCS experience only one or a few OCS and will never experience impairment or significant distress from them, approximately 10–20% meet criteria for what would be considered to be clinically relevant but subthreshold symptoms. That is, these individuals experience more than occasional OCS, and some mild distress associated with them, but their symptoms do not rise to the level of severity to be considered clinically actionable. A clinical diagnosis of OCD may therefore represent a more severe manifestation (i.e. the extreme end) of a much broader continuous phenotype as is expected under a liability threshold model (Fig. 1).

If this model is correct, including population-based samples of participants with some level of OCS in genetic studies would increase the available sample size and thus the power to detect genetic variants. However, this approach assumes that the OCS phenotype being used (typically OCS counts based on self-report questionnaires) indeed reflect the underlying genetic liability and hence correlate with OCD as clinically defined. But what is the evidence that this is true? In addition to determining whether OCS are in fact heritable, it is important to define the genetic relationships between the dimensional (i.e., OCS count, OCS severity, etc.) and categorical (i.e., OCD diagnosis) traits. There are two commonly used ways to determine if such a relationship exists. The first is to use polygenic risk score (PRS) analysis, which is a single value estimate of an individual’s SNP-based genetic liability in one trait or disease, to estimate the relative risk of having the second trait based on its SNP-based genetic liability profile. The second is genetic correlation analysis, which estimates the proportion of variance that two traits share based on their genetic similarity.

#### Heritability of OCS

Twin studies indicate that, like OCD, subclinical OCS are heritable, with genetic factors accounting for 36–42% of its phenotypic variance [75, 76]. Similarly, individuals with OCD tend to score high on the quantitative measures used to assess OCS (although this is not universally the case) [77]. But, while the nature of OCS as a phenotypic continuum, and the heritability of symptoms along this continuum, has been appreciated clinically for many years, only recently has a comprehensive picture based on genotypic data emerged, indicating the possibility that an underlying genetic liability spectrum may, in part, account for this distribution. To date, three GWAS have examined the SNP-based or genomic heritability of OCS in population-based samples and its genetic relationship to OCD [23, 25, 75]. As predicted by the liability spectrum model, the SNP-based heritability estimates of OCS are lower than the heritability estimates derived from twin and family studies, and also lower than SNP-based heritability estimates for OCD. Reported heritability estimates for OCS range from 6.8% in Burton et al. [23], who quantified OCS using the standardized total score of the average amount of time spent on obsessive-compulsive behavior reported on the Toronto Obsessive-Compulsive Scale (TOCS) questionnaire, (SE = 0.051, *p* = 0.19, *N* = 5,018), to 10% in the Smit et al. sample (SE = 0.06, *p* = 0.095, *N* = 8,267) [78], and 14% in the den Braber et al. sample (SE = 0.05, *p* = 0.003, *N* = 6,931) [75], the latter two studies using the total score of an abbreviated version of the self-report Padua Inventory Revised and the Padua Inventory, respectively.

#### Genetic relationship between OCS and OCD

If OCD and OCS are indeed part of the same genetic and phenotypic continuum, one would expect to see significant genetic correlation between OCS and OCD. These relationships have been explored using a variety of methods including linkage disequilibrium regression score (LDSC) analysis. LDSC analysis allows the use of summary results in tests of genetic correlation (see Box). Burton et al. [20] reported a high but statistically underpowered genetic correlation between OCD and OCS (*r*_*G*_ = 0.83, SE = 0.43, *p* = 0.073) [23], whereas Smit et al. did not find a genetic correlation between OCD and OCS as a general measure (incorporating both obsessive and compulsive symptoms jointly) but did find a significant genetic correlation between OCD and one of two subscale measures of OCS (the compulsion subscale; *r*_*G*_ = 0.61, *p* = 0.017) [78].

Polygenic risk score (PRS) (see Box) analyses performed in the three population samples discussed above provide an alternative approach to test the genetic correlation between subclinical OCS and clinical OCD. Using this method, PRS trained on genetic associations from OCS in the Burton et al. [20] sample were associated with OCD case/control status in an independent sample of OCD cases and controls (Nagelkerke’s pseudo *R²* = 0.28%, *p* = 0.0045) [23]. This association also held true when reversing the analysis and predicting population-based OCS using PRS trained on associations from OCD case/control GWAS studies. PRS trained on OCD case/control GWAS explained between 0.2% and 0.57% of the phenotypic variance in each of the population-based OCS, depending on the sample and on the thresholds used in the analysis [23, 75, 79] Though the proportion of phenotypic variance explained is very small, these findings indicate that OCD and OCS share some genetic associations and suggest that as sample sizes grow, so too will the evidence for genetic correlation between OCD and OCS. In summary, genetic risk for OCD susceptibility, calculated as SNP-based aggregated genetic risk, appear to contribute to OCS, and genetic risk for OCS calculated in the same way appears to contribute to OCD.

Only two GWAS studies to date have examined the unique and shared genetic architecture of OC symptom factors, one of which used data from clinical OCD patients [22], and the other of which used data collected in the general population [25]. These studies, while suggesting some genetic sharing between symptom subtypes, still require replication. Bralten et al. observed significant genetic sharing between OCD and one symptom dimension, ‘guilty taboo thoughts’, in the general population using PRS analysis (*N* = 650, *R²* = 2.28%, *p* = 0.002) [25], and after combining their data with OCS data from another population-based OCS GWAS [23] they also reported significant genetic sharing between OCD and the ‘symmetry/counting/ordering’ dimension (*R²* = 0.49%, FDR-adjusted *p* = 2.42 × 10^−5^) as well as the ‘contamination/cleaning’ dimension (*R²* = 0.23%, FDR-adjusted *p* = 4.07 × 10^−3^). An independent pathway analysis by Burton et al. [24] showed that additive genetic effects accounted for most of the shared variance across symptom subtypes, whereas subtype-specific variance was mostly explained by unique environment, with an exception for hoarding and superstition, for which subtype-specific variance was accounted for by additive genetic factors [24]. In other words, this study suggests that what makes the OC dimensions similar is shared genetic factors and what makes them different is unique environmental factors, consistent with earlier findings suggesting that a single unidimensional obsessionality construct accounts for the majority of heritability in OCS identified in family studies [65]. So far, only two genome-wide significant SNPs have been identified as associated with OC symptom subtypes [23, 75, 78], which still await replication.

#### Comparison of the dimensional versus diagnostic framework

These studies, although not conclusive, do suggest that subthreshold obsessive-compulsive symptoms share genetic risk with OCD. However, even among individuals who endorse no OC symptoms, there may be appreciable genetic risk for OCD, suggesting statistical power for studies invoking a dimensional framework remains a concern (Fig. 1). Moreover, the ways in which OC symptoms are assessed and measured may also impact their observed genetic relationship with OCD. For example, distress or impairment are inherently ascertained in studies employing a diagnostic framework, making them inseparable from other aspects of OCD (i.e. number of symptoms, symptom type), but are not necessarily ascertained or measured in studies employing a dimensional framework. Thus, the choice of symptom dimension (e.g., number of symptoms or degree of distress associated with symptoms) or the way in which symptoms are assessed or defined may yield differing genetic correlations and insights when compared to clinically ascertained OCD.

### Relationships between OCD and other psychiatric diagnoses

#### Phenotypic correlations

The heterogeneity, and perhaps also the dimensionality associated with OCD, can also be seen in the phenotypic overlap and comorbidity patterns with other psychiatric disorders, and in particular, with the Obsessive-Compulsive Related Disorders (OCRDs [80, 81]; see Fig. 3). The idea that OCD is part of a cluster of disorders with obsessive and/or compulsive symptoms as a core feature was acknowledged in the most recent version of the Diagnostic and Statistical Manual of Mental Disorders (DSM-5). In the DSM-5, unlike in previous editions, OCD is no longer considered an anxiety disorder but rather is the flagship diagnosis for a newly created group of disorders, called the Obsessive-Compulsive and Related Disorders (OCRD). Other disorders in this category include: hoarding disorder (HD), which affects approximately 4% of the population and is more common in older adults [82], body dysmorphic disorder (BDD) which affects approximately 2.4% of the population [83, 84], trichotillomania (TTM), with a point prevalence of 0.5–2.0% of the population and with peak age of onset between the ages of 10–13 [85, 86], and excoriation disorder (ExD) which affects approximately 3% of the population [87]. While tic disorders such as Tourette Syndrome (TS) and Persistent Motor or Vocal Tic Disorder (PMVT) are not included in the DSM-5 OCRD category, tic-related OCD is a specifier in the DSM-5 criteria for OCD, acknowledging the strong etiological and clinical comorbidity (30–50%) [14,21,88,89,] between these disorders.

Among individuals with OCD, the most common comorbid diagnoses are anxiety disorders (ANX ~76%) [1, 90], mood disorders (~63%) [1, 90], particularly major depressive disorder (MDD, ~40%) [1, 91, 92], impulse-control disorders (~56%) [1, 93, 94], substance use disorders (SUD, ~39%) [1], Tourette syndrome (TS, 10-12%) [88, 89], and anorexia nervosa (AN, 5-10%) [95, 96]. OCD also frequently co-occurs with neurodevelopmental conditions including autism spectrum disorders (ASD, 17% [97, 98]) and attention-deficit hyperactivity disorder (ADHD, ~21% in childhood OCD, 8.5% in adults) ADHD [1,91,99,100,]. In addition to being highly comorbid, OCD also shares phenotypic characteristics with some of these neuropsychiatric disorders. For example, repetitive behaviors are a core feature of ASD, and are observed in tic disorders. In OCD, these behaviors are called compulsions, in TS they are called complex tics, and in ASD they are called stereotypies or restricted repetitive behaviors. Although there are operationalized distinctions between these symptoms (e.g., presence (or lack of) other symptoms such as distress or accompanying anxiety, apparent soothing quality, or apparent lack of control) in practice, they can be difficult to differentiate, particularly by those who are not experts in the diagnosis of the disorder in question. On the one hand, this limitation may also impact studies of genetic correlation across disorders, particularly if diagnoses are confounded by these overlapping symptom patterns. However, on the other hand, this transdiagnostic feature of many disorders provides an avenue for further cross-disorder dimensional analyses.

For example, although genetic correlations between the OC dimensions and other psychiatric disorders have not yet been examined, some work has been done to assess the phenotypic correlations between these symptom subtypes and psychiatric illness. Hirschtritt et al [101] systematically characterized the relationship between psychiatric comorbidities and eight OC dimensional symptom subtypes derived from factor analysis in a large number of individuals with TS and their family members (*N* = 3494). All eight symptom dimensions were highly heritable in this sample (*h*^2^ from 0.20 to 0.37, *p* values <10^−15^). They found that TS was correlated with symmetry/exactness, aggressive obsessions, and fear of harm obsessions, anxiety disorders were correlated with contamination/cleaning symptoms, disruptive behavior disorders were associated with aggressive obsessions and correlated with disruptive behavior disorders, and ADHD was correlated with hoarding obsessions/compulsions and with aggressive obsessions. Other studies corroborate relationships between OCD symptom dimensions and psychiatric comorbidities, most consistently with aggressive obsessions and hoarding symptoms, which are associated with multiple psychiatric illnesses in several studies [102–104]. This approach, while providing additional information, has yet to identify consistent relationships between psychiatric illnesses and OC symptom subtypes. This is in part due to the fact that the symptom dimensions are not consistently defined, but rather are derived from sample-specific factor analyses (numbers of dimensions ranged from four to eight). Similarly the genetic correlations between psychiatric illnesses and obsessive-compulsive symptoms have not yet been assessed.

#### Genetic relationships

Twin and family studies indicate that many of these conditions are not only highly comorbid, but are also heritable [105–108] and appear to share genetic etiologies with OCD [12, 76, 109–112].

Few studies have examined the genetic relationships between the OCRDs, partly because several of these disorders were not recognized as distinct illnesses until their inclusion in the DSM-5 in 2013. However, one such study that used multivariate twin modeling to examine genetic relationships between quantitative symptom-based measures of the OCRDs identified two primary latent factors, one of which loaded significantly on all five OCRD phenotypes (OCD, HD, BDD, TTM, and ExD), but most strongly on OCD, HD, and BDD, while the other was specific to the body focused repetitive behavior (BFRB) disorders (TTM and ExD). The additive genetic variance was 63.3% (95% CI, 59%-66) for the first (common) factor, and 73.7% (95% CI, 30–99%) for the second, BFRB specific factor [106]. Two twin studies (*N* = 7567 [76] and *N* = 5293 [113]) that examined the genetic relationships between OCD and HD using quantitative measures also found significant genetic contributions to both OCS (40%) and hoarding symptoms (36%), with reported genetic correlations of HD/hoarding symptoms and OCS between 0.10 [76] and 0.41 [113].

Of the non-OCRD psychiatric or neurodevelopmental disorders that have been examined in conjunction with OCD, AN and TS have the most consistent and strongest evidence for genetic correlations with OCD. The genetic correlation between OCD and AN is around 0.53 [80, 114, 115], although a cross-disorder GWAS of OCD and AN did not identify genome-wide significant SNPs associated with these disorders. Similarly, the genetic correlation between TS and OCD is consistently estimated to be around 0.4 [50, 80, 114], meaning that on average, about 40% of the risk alleles relevant for OCD are also relevant for TS. However, cross-disorder GWAS of OCD and TS has not yet identified genome-wide significant variants.

The possible shared genetic basis of OCD and other neuropsychiatric disorders has also recently been a focus of attention [80, 81, 116, 117]. Cross-disorder GWAS examining the common and unique genetic underpinnings of eight psychiatric disorders, including OCD, found that all of the disorders share a substantial proportion of their common variant genetic risk, with many of the associated variants exercising pleiotropic (i.e. diverse) effects on more than one disorder [80, 81]. In another cross-disorder GWAS example, examining OCD, TS, ASD, and ADHD, Yang et al. identified 297 SNPs that were significantly associated with OCD, TS, ASD, or ADHD [118]. Of these, 177 SNPs showed the same direction of effect across all traits, suggesting that they contributed to genetic risk of all four disorders and 199 SNPs demonstrated a high probability of association with at least three out of the four disorders.

**Fig. 4:**
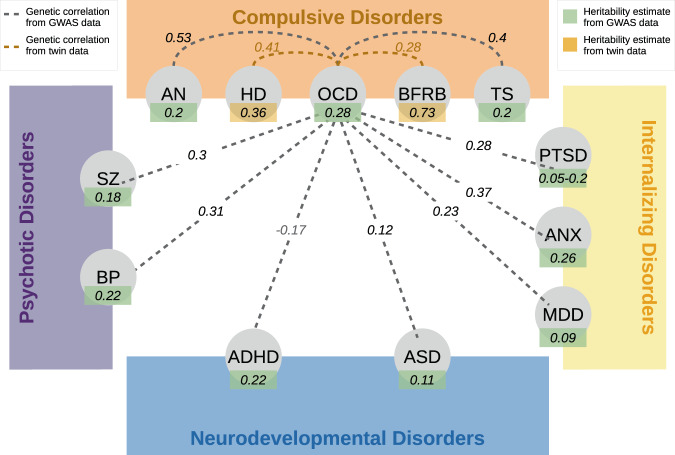
Heritability estimates and genetic correlation estimates between OCD and related psychiatric disorders. For SZ, MDD, BP, ADHD, TS, AN, ANX, and PTSD heritability estimates (green boxes) and genetic correlations (black lines) were calculated using genome-wide data [80, 81, 117, 118, 121]. Whereas heritability estimates (pink boxes) and genetic correlations (orange lines) for HD [76, 113] and BFRBs [106] were calculated using twin-data.

Factor analysis as implemented in genomic structural equation modeling (Genomic SEM) [34], which models the latent genetic structure across psychiatric disorders [81, 116, 117], has shown that OCD, AN, and TS consistently cluster together [117] in what has been termed a “compulsive disorders factor” [81] or a “compulsive/perfectionist behaviors factor” [116], the latter also including a significant loading onto MDD. This compulsive factor also demonstrates high correlations with other factors identified through Genomic SEM, including an internalizing disorders factor (alcohol dependence (ALC), posttraumatic stress disorder (PTSD), MDD, and ANX), psychotic disorders factor (SCZ, bipolar disorder (BIP), ALC), and to a lesser degree, a neurodevelopmental disorders factor (ADHD, ASD, PTSD, and MDD) [81]. The patterns of genetic correlations between the individual disorders in the compulsive disorders factor and various biobehavioral traits indicated that in addition to some shared genetic architecture, OCD, AN and TS also show unique genetic patterns of association. These findings are corroborated by other methods [80, 114, 115] but it is important to note that almost all studies investigating genome-wide genetic sharing between disorders use the same publicly available data [119]. OCD also shows high bivariate genetic correlations with almost all of the psychiatric disorders, such as MDD [80, 81], ASD [80, 117, 118, 120], SZ [80, 117, 121], BP [80, 81, 117] with limited evidence for a negative correlation with ADHD [80, 117, 118] (Fig. 4). An ADHD-OCD cross-disorder GWAS reported that of the overlapping SNPs identified, one third showed opposite direction of effects for OCD and ADHD, indicating that they predisposed to one disorder and were protective for the other [118].

Although OCD was originally classified as an anxiety disorder, and anxiety is a prominent symptom, OCD is now recognized as a disorder with biological and clinical characteristics that are distinct from anxiety disorders. Nevertheless, these disorders are highly comorbid, and thus there is continuing interest in determining whether they share a genetic etiology. Genetic studies of anxiety disorders are still in the early stages, and data on the correlation between the anxiety disorders and OCD are also limited. While the point estimates for genetic correlations between OCD and anxiety disorders (*r*_*G*_ = 0.37, SE = 0.21, *p* = 0.07, [80]), and PTSD, another psychiatric illness once classified as an anxiety disorder and now categorized separately (*r*_*G*_ = 0.28, SE = 0.20, *p* = 0.13 [80]) are high, these correlations do not yet reach statistical significance, most likely due to small sample sizes, particularly for the OCD samples.

In sum, while the degree and nature of the genetic relationship between OCD and related disorders are yet to be determined with precision, even these early results necessitate an open-minded approach to the nosology of OCD guided by genetics (Fig. 4). Certainly, increasing sample sizes for all discovery efforts will be critical to dissect the genetic relationships between OCD and its constellation of comorbidities. Newly developed statistical methods such as stratified Genomic SEM [34] or Case/-Case-GWAS [122], a method that tests for differences in allele frequency among cases of two different disorders, will also aid future analyses that try to disentangle the shared and unique genetic basis of psychiatric disorders and their specific correlates. But perhaps most exciting is the potential for applying the dimensional framework in future genomic studies to capitalize on phenotypic measures that may cross diagnostic boundaries. For example, neuropsychological and other studies indicate that OCD and related disorders vary along what can be conceived as continuous, biobehavioral and psychological dimensions, including, but not limited to domains involving cognition and beliefs (e.g., obsessions in the case of OCD), habit and behavior (e.g., compulsions), reward and reinforcement (e.g., relief from anxiety or distress caused by obsessions), response inhibition and error monitoring (e.g., intolerance of uncertainty inherent in obsessional thinking), and neuroticism and emotion sensitivity (e.g., distress tolerance). Each of these dimensions provides a mechanism for transdiagnostic phenotyping that can be investigated with genetic studies and compared to existing genomic data to further resolve these relationships and understand the path from genomic variation to clinical impairment.

## Conclusion

There is now an emerging body of molecular evidence to suggest that OCD shares genetic features with many other psychiatric disorders and with subclinical OCS in the population. Nevertheless, it is clear that increased sample sizes and creative approaches to phenotypic data collection will be necessary to move the field forward. OCD and its related psychiatric disorders appear to vary along continuous, biobehavioral, and psychological dimensions, including, but not limited to, cognition and beliefs, habit and behavior, reward and reinforcement, error monitoring response inhibition, as well as neuroticism and emotion sensitivity. It has been suggested that genetic variants responsible for susceptibility to mental illness may not always line up with current diagnostic categories, and there are initiatives to identify shared dimensional phenotypes that “lie in between” diagnostic labels and risk genotypes [123–127]. Dissecting how each disorder can be conceptualized, phenotypically and genetically, with regards to each dimension, will likely guide future research in psychiatric genomics. In the future, shared heritable behavioral and psychological correlates probing transdiagnostic dimensions will likely shed more light on the genetic underpinnings of OCD, the high comorbidity rates for OCD, and genetic correlations that we observe between OCD and related psychiatric disorders.
